# Effects of Smoking Cessation on Epigenetic Aging

**DOI:** 10.1093/geroni/igab046.2524

**Published:** 2021-12-17

**Authors:** Brian Chen, Weiye Wang, Nichole Rigby, Randal Olson, Steve Sabes

**Affiliations:** 1 FOXO Technologies, Minneapolis, Minnesota, United States; 2 FOXO Technologies, FOXO Technologies, California, United States; 3 FOXO Technologies, FOXO Technologies, Minnesota, United States

## Abstract

“Epigenetic clocks” have become widely used to assess individual rates of biological aging. However, experimental data are limited in humans to identify potential confounding factors that may influence one’s rate of epigenetic aging and multiple health outcomes. We examined multiple epigenetic aging measures among regular smokers who quit smoking for two weeks. DNA methylation markers were assessed in both whole blood and saliva at multiple time points using a customized DNA methylation microarray. Generally, no changes in epigenetic aging rates were detected in the two week observation period with the exception of pronounced decreases over time in rate of Hannum’s clock and Extrinsic Epigenetic Age Acceleration in blood DNA. In saliva DNA, decreases over time were detected in the rates of the GrimAge and DNAmPhenoAge clocks, but we saw an increase in the rate of the Skin and Blood Clock. Additional experimental studies of other common exposures may be useful to better characterize factors that may affect the observed “rate” of epigenetic aging.

